# Supplementary material for: Prospective accuracy study on an artificial intelligence-based ultrasound system for gestational age estimation among pregnant women in Ghana, Kenya and South Africa: protocol
Source: BMJ Open. 2026 Sep 11;16(9):e118330. doi: 10.1136/bmjopen-2026-118330 (PMC13583877; doi:10.1136/bmjopen-2026-118330)
Supplement: Supplementary file 1 [file bmjopen-2026-118330.reviewer_comments.pdf]

PEER REVIEW HISTORY

BMJ Open publishes all reviews undertaken for accepted manuscripts. Reviewers are asked to complete a checklist review form and are provided with free text boxes to elaborate on their assessment. These free text comments are reproduced below.

ARTICLE DETAILS

Title (Provisional)

Protocol for a prospective accuracy study on an artificial intelligence-based ultrasound system for gestational age estimation among pregnant women in Ghana, Kenya and South Africa

Authors

Swarray Deen, Alim; McDougall, Annie; Chemway, Rosa; Craik, Rachel; Jayaratnam, Skandarupan; Joseph, Naima; Mahar, Robert; Koye, Digsu; Nguyen, Long; Simpson, Julie; Gwako, George; Hadebe, Ruth Lorna; Nartey, Edmund Tetteh; Minckas, Nicole; Gülmezoglu, Ahmet Metin; Vogel, Joshua Peter; Osman, Ayesha; Collaborators, PEARLS

VERSION 1 - REVIEW

**Reviewer**	**1**
**Name**	**England, Andrew**
**Affiliation**	**University College Cork**
**Date**	**26-Jul-2026**
**COI**	**None**

I commend the authors for this protocol manuscript. Given that this study has not yet been completed, there are limited areas in which I can comment.

1. Page 5 - apologies I did not understand the term 'in silico'.

2. I would be interested to see further explanation of the term accredited sonographer. There are very few universal standards for sonography practice. Thus training and accreditation could very within and between countries.

3. Page 7 - why capitalise Consultants?

4. How will you ensure suitable BMI variation amongst participants?

5. I would have expected to see more details regarding the trained midwives and the sweep process / protocol.

6. In terms of ethics are US images upload to the 'cloud', are participants particularly consented about the AI elements?

7. Was the AI platform trained on African datasets?

I would ask the Journal to consider whether Protocols fit within the 'scope' of the Journal. There are specific protocol journals available - I think.

VERSION 1 - AUTHOR RESPONSE

Reviewer comments	Response
1. Page 5 - apologies I did not understand the term 'in silico'.	An explanation of the term "in silico" has been added to the text:<br><br>Page 5, line 190: in silico (retrospective computer-based validation).
2. I would be interested to see further explanation of the term accredited sonographer. There are very few universal standards for sonography practice. Thus training and accreditation could very within and between countries.	All sonographers were accredited according to ISUOG standards. This has now been clarified in the methods section: Page 8, Lines 274-275.
3. Page 7 - why capitalise Consultants?	We have removed the capitalisation.
4. How will you ensure suitable BMI variation amongst participants?	Thank you for this comment. The study did not include BMI-based eligibility criteria or recruitment quotas. Participants were recruited consecutively from routine antenatal care populations across multiple sites and countries, with the aim of reflecting the intended real-world population in which the technology would be used. Based on previous cohorts conducted in these same settings, we anticipated recruitment across a broad range of maternal BMI values.* Our previous cohort included women across the underweight, normal weight, overweight and obese BMI categories, providing confidence that an all-comers recruitment strategy would capture substantial BMI variability. Maternal BMI was collected for all participants and will be examined in exploratory subgroup analyses to assess whether device performance varies according to BMI.<br><br>*https://papers.ssrn.com/sol3/papers.cfm?abstract_id=6681363
5. I would have expected to see more details regarding the trained midwives and the sweep process / protocol.	Thank you for this suggestion. The sweep acquisition procedure is standardised through device-based video instructions and is illustrated in Figure 2. To further clarify the procedure, we have added additional detail to the Methods section specifying that the acquisition consists of six abdominal sweeps: three sweeps from the lower abdomen to the upper abdomen followed by three sweeps from left-to-right across the abdomen. As the procedure is guided in real time by

	the device and requires minimal operator decision-making, we believe the revised description and accompanying figure provide sufficient detail for replication of the protocol.
6. In terms of ethics are US images upload to the 'cloud', are participants particularly consented about the AI elements?	We thank the reviewer for this comment. Ultrasound images acquired using the IU ScanNav FetalCheck device are routinely uploaded to a secure cloud storage service managed by Intelligent Ultrasound/GE Healthcare for quality assurance and performance monitoring of the device, as specified in a Data Sharing Agreement between the study sponsor and the company. Only a unique study participant identification number accompanies the uploaded images; no directly identifying information is shared, and the company does not have access to information required to re-identify participants. Participants are informed of this data sharing arrangement during the consent process, and this information is also described in the Participant Information and Consent Form.<br><br>Participants are informed that the study is evaluating a novel software-based ultrasound tool for gestational age estimation. As no participant data are used for AI model development, additional consent for algorithm training was not required.
7. Was the AI platform trained on African datasets?	As specified in the introduction, the AI platform was not trained on African datasets (lines 188-196). One of the primary objectives of the present study was therefore to prospectively evaluate the performance of the platform in African populations and under real-world implementation conditions within routine, high-volume antenatal care settings. The study was designed to provide external validation of the algorithm in populations and healthcare environments that were distinct from those used during model development.
